# Prevention of Post-ERCP Pancreatitis

**DOI:** 10.1155/2012/796751

**Published:** 2011-08-10

**Authors:** F. Donnellan, Michael F. Byrne

**Affiliations:** Department of Gastroenterology, Division of Medicine, Vancouver General Hospital, Vancouver, BC, Canada V5Z 1M9

## Abstract

Pancreatitis is the most common complication of ERCP. It can be associated with substantial morbidity. Hence, the minimization of both the incidence and severity of post-ERCP pancreatitis is paramount. Considerable efforts have been made to identify factors that may be associated with an increased risk of this complication. In addition, both procedure- and pharmacological-related interventions have been proposed that may prevent this complication. This paper outlines these interventions and presents the evidence to support their use in the prevention of post-ERCP pancreatitis.

## 1. Introduction

The prediction of post-ERCP pancreatitis is difficult. However, a number of factors have been identified that place patients at a relatively higher risk. These include both patient and procedure-related factors. A number of procedure-related interventions have been proposed that may reduce the risk of pancreatitis. Furthermore, identification of the mechanism of injury and the subsequent cascade of events leading to the clinical manifestation of pancreatitis has also resulted in the use of pharmacological interventions to reduce the risk of this complication.

This paper describes both the procedure- and pharmacological-related interventions currently being proposed for use in the prevention of post-ERCP pancreatitis.

## 2. Diagnosis of Post-ERCP Pancreatitis

Post-ERCP pancreatitis is defined as acute pancreatitis that has developed de novo following ERCP and, based on consensus guidelines proposed by Cotton et al. in 1991, is the presence of new pancreatic-type abdominal pain associated with at least a threefold increase in serum amylase concentration occurring 24 hours after an ERCP, with pain severe enough to require admission to the hospital or to extend an admitted patient's length of stay [1]. 

The severity of post-ERCP pancreatitis is mainly based on the length of hospitalization: mild post-ERCP pancreatitis is defined as need for hospital admission or prolongation of planned admission up to 3 days, moderate post-ERCP pancreatitis as need for hospitalization of 4–10 days, and severe post-ERCP pancreatitis as hospitalization for more than 10 days, or hemorrhagic pancreatitis, pancreatic necrosis, or pseudocyst, or need for percutaneous drainage or surgical intervention.

## 3. Incidence of Post-ERCP Pancreatitis

Most studies reporting ERCP complications have specifically analyzed the risk associated with sphincterotomy. Freeman et al. demonstrated an overall incidence of post-ERCP pancreatitis of 5.4% following endoscopic biliary sphincterotomy in a multicentre prospective study of 2347 patients involving 17 centers, [2]. Based on consensus guidelines previously discussed [1], pancreatitis was graded as mild in 42%, moderate in 51%, and severe in 7% with a mortality rate of 0.8%. Pancreatitis was also found to be the most frequent complication occurring in 3.5% of cases in a systematic review of 21 studies involving 16,885 patients undergoing unselected ERCP (both diagnostic and therapeutic). It was graded as mild in 45%, moderate in 44%, and severe in 11% of cases with a mortality rate of 3% [3].

## 4. Mechanisms of Post-ERCP Pancreatitis

A number of mechanisms have been proposed as potential triggering factors in the development post-ERCP pancreatitis. Mechanical injury to both the papilla and pancreatic duct may occur in response to instrumental manipulation resulting in impaired drainage from the pancreas. Thermal injury may develop following application of electrosurgical current during biliary or pancreatic sphincterotomy. Chemical injury may result following injection of contrast medium into the pancreatic duct. Hydrostatic injury may result following injection of contrast medium into the pancreatic duct or from infusion of water or saline solution during sphincter manometry. Irrespective of the mechanism, the initial injury leads to a cascade of event resulting in the premature activation of proteolytic enzymes, autodigestion, and impaired acinar secretion with subsequent clinical manifestations of local and systemic effects of pancreatitis. Most approaches to the prevention of post-ERCP pancreatitis are aimed at interruption of one of the points in this cascade.

## 5. Risk Factors for Post-ERCP Pancreatitis

It is important to identify cases in which there is a relatively higher risk of pancreatitis so that preventive measures such as pancreatic stenting or pharmacological prophylaxis may be considered. Assessment of both patient- and procedure-related factors is important to determine such high-risk cases (Table 1). Masci et al. in a meta-analysis of 15 studies identified three patient-related and two procedure-related factors associated with a definite risk of post-ERCP pancreatitis. The patient-related factors included suspected sphincter of Oddi dysfunction (relative risk (RR) 4.09, 95% CI 3.37–4.96; *P* < 0.001), female gender (RR 2.23, 95% CI 1.75–2.84; *P* < 0.001), and previous pancreatitis (RR 2.46, 95% CI 1.93–3.12; *P* < 0.001). The procedure-related factors included precut sphincterotomy (RR 2.71, 95% CI 2.02–3.63; *P* < 0.001) and pancreatic injection (RR 2.2, 95% CI 1.6–3.01; *P* < 0.001) [4].

Additionally, multiple attempts (greater than 10 attempts) at cannulation (odds ratio (OR) 14.9, 95% CI 10.50–21.26; *P* < 0.001), pain during ERCP (OR 1.9, 95% CI 1.113–3.438; *P* = 0.01) [5], minor papilla sphincterotomy (OR 3.82, 95% CI 2.003–7.106; *P* < 0.0001), age < 60 years (OR 1.61, 95% CI 1.33–2.402; *P* = 0.04), ⩾2 contrast injections into the pancreatic duct (OR 1.5, 95% CI 1.046–2.103; *P* = 0.03), trainee involvement (OR 1.5, 95% CI 1.029–2.057; *P* = 0.03) [6], moderate to difficult cannulation (6 to greater than 15 attempts) (OR 3.41, 95% CI 2.13–5.47; *P* = 0.0001), pancreatic sphincterotomy (OR 3.07, 95% CI 1.64–5.75; *P* = 0.0001), a normal serum bilirubin (OR 1.89, 95% CI 1.22–2.93; *P* = 0.0023), and absence of chronic pancreatitis (OR 1.87, 95% CI 1.00–3.48; *P* = 0.0471) [7] have all been shown by multivariate analysis to be risk factors for post-ERCP pancreatitis. Furthermore, risk factors are likely to be cumulative so that patients with multiple factors are at an extremely high risk of developing pancreatitis [7].

## 6. Prevention of Post-ERCP Pancreatitis

### 6.1. The Endoscopist

#### 6.1.1. Case Volume

The indications for ERCP are likely to be different in low volume compared with high-volume centers and hence might impact on the reported rates of pancreatitis. High-volume centers have been shown to perform a significantly larger number of more difficult procedures in patients at an increased risk of pancreatitis [5].

However, there is no evidence that ERCP case volume influences the rate of post-ERCP pancreatitis. Both Williams et al. [8] and Testoni et al. [5] demonstrated in prospective multicentre studies that the risk of pancreatitis was not associated with either the case volume of the single endoscopist or the center. In contrast, trainee participation has been shown to be a significant risk factor for the development of post-ERCP pancreatitis [6].

*The incidence of post-ERCP pancreatitis is not dependent on the case volume of the endoscopist or the center.*

### 6.2. ERCP Techniques

#### 6.2.1. Standard Cannulation

The standard method of biliary cannulation at ERCP utilizes a catheter device with or without a soft tip guidewire. Contrast injection through the catheter can also facilitate deep cannulation of the common bile duct. However, inadvertent contrast injection of the pancreatic duct may occur. In contrast, with guidewire cannulation, entry into either the bile or pancreatic duct is determined by fluoroscopy obviating the need for contrast injection and possible pancreatic duct filling. 

While a large randomized controlled trial by Bailey et al. involving 413 patients failed to show a difference in pancreatitis between the two approaches (7.9% in the guidewire group versus 6.2% in the contrast group; *P* = 0.48) [9], a number of studies, with similarly large patient sizes, demonstrated a lower rate with guidewire cannulation (8.6% versus 16.6%; *P* = 0.037 [10], 2.0% versus 11.3%; *P* = 0.001 [11]).

Furthermore, Cheung et al. concluded from a systematic review of 7 randomized controlled trials totaling 2132 patients that guidewire cannulation significantly reduced the risk of pancreatitis compared with contrast injection (3.2% versus 8.7%; RR 0.38, 95% CI 0.19–0.76) [12]. 

*The wire-guided technique is recommended for biliary cannulation.*

#### 6.2.2. Pancreatic Duct Injection

Pancreatic duct injection and in particular multiple injections are a risk factor for post-ERCP pancreatitis development [13]. As already mentioned, Cheng et al. found in a prospective multicentre study involving 15 US centers and 1115 patients that two or more contrast injections of the pancreatic duct were significantly associated with the development of pancreatitis [6]. Furthermore, Cheon et al. demonstrated in a retrospective study that a higher rate of pancreatitis was associated with any pancreatic duct opacification compared with bile duct opacification alone (6.9% versus 0.8%, *P* = 0.001) and an increased extent of duct opacification (head only versus head and body versus head, body, and tail) (3.6% versus 4.5% versus 8.6%) [14]. ERCP is being increasingly used in the diagnosis of pancreatic cystic neoplasms, in particular, to determine communication of the cyst with ductal system. If a pancreatogram is required in such circumstances, or indeed occurs inadvertently, it is recommended to keep the number of injections and the volume injected to a minimum [15].The mechanism by which contrast injection can cause pancreatitis remains controversial. The osmolality of the contrast media used has been proposed as a possible contributing factor. Low-osmolality is thought to be safer than high-osmolality contrast media as it is associated with less osmotically driven fluid shifts and subsequent lower increases in intraductal pressure. While the results from a number of randomized trials have been contradictory [16, 17], the meta-analysis by George et al. showed that there was no significant difference between high- and low-osmolality contrast media with respect to the development of pancreatitis [18].

*Pancreatic duct injection, if occurs inadvertently or required, should be kept to a minimum.*

#### 6.2.3. Pancreatic Guidewire-Assisted Biliary Cannulation

Pancreatic guidewire placement can be effectively used to facilitate biliary access, by straightening the ampulla and preventing pancreatic duct cannulation. This technique has been used in selected cases of difficult biliary cannulation where the pancreatic duct is unintentionally cannulated repeatedly and relatively easily [19]. Two randomized controlled studies comparing this technique with continuing standard cannulation have produced conflicting results regarding the development of post-ERCP pancreatitis. In the study by Maeda et al., no cases of pancreatitis were identified in 53 randomized patients. Furthermore, no pancreatic stents were placed [20]. In contrast, Herreros de Tejada et al. demonstrated a nonstatistically significant higher rate of pancreatitis in the pancreatic guidewire group (97 patients) compared with the standard cannulation group (91 patients) (17% versus 8%; *P* = 0.079) [21]. 12 out of 97 patients in the pancreatic guidewire group in this latter study underwent pancreatic stenting. The question of whether pancreatic stenting is required subsequent to guidewire placement was addressed in a randomized controlled study by Ito et al. They found a significantly lower risk of pancreatitis in 35 patients in whom a pancreatic stent (5 French 4 cm single pigtail) was inserted following guidewire placement compared to the same number of patients in whom no stent was inserted (2.9% versus 23%; RR 0.13, CI 0.016–0.95) [22].

*Pancreatic duct stenting after guidewire placement for achieving selective biliary cannulation is recommended to reduce the incidence of post-ERCP pancreatitis.*

#### 6.2.4. Pancreatic Duct Stenting

Impaired drainage of the pancreatic duct, resulting from papillary edema or spasm of the sphincter of Oddi, has been proposed as a cause or a risk factor for the development of post-ERCP pancreatitis. This has resulted in placement of pancreatic duct stents in high-risk cases in an effort to prevent post-ERCP pancreatitis. However, there is no consensus as to exactly which cases merit stent placement.

A number of prospective randomized trials have demonstrated the benefit of pancreatic stent insertion in reducing both the rate and severity of post-ERCP pancreatitis after difficult cannulation, needle-knife precut, biliary sphincterotomy for sphincter of Oddi dysfunction (SOD) and manometry, pancreatic sphincterotomy, and endoscopic balloon dilation [23–31] (Table 2). The recent meta-analysis by Choudhary et al. further confirmed these results demonstrating that prophylactic pancreatic stent placement significantly decreased the odds of post-ERCP pancreatitis (OR, 0.22; 95% CI, 0.12–0.38; *P* = 0.01) [32].

Pancreatic stents are not without problems. Follow-up evaluation is necessary to ensure passage or removal. In addition, placement can be technically difficult. Smithline et al. and Aizawa and Ueno found that stent placement was unsuccessful in 5 out of 48 patients (10.4%) and 2 out of 40 patients (5%), respectively [23, 28]. Furthermore, unsuccessful stent placement can itself be associated with a risk of pancreatitis. A prospective study of 225 high risk ERCPs by Freeman demonstrated that pancreatitis developed in 2 out of 3 patients (66.7%) in whom pancreatic stenting failed, compared to 32 out of 222 (14.4%) in whom stenting was successful (*P* = 0.06). Interestingly, stent placement was unsuccessful in 3 of the 93 cases in which conventional deep guidewire insertion into the pancreatic duct was used compared with none of the 132 cases in which a modified technique involving an 0.018-inch guidewire, passed as little as 1 to 2 cm beyond the pancreatic sphincter, was used [33].

There is wide variation in both the guidewire and the type of stent used for prophylaxis of post-ERCP pancreatitis. Brackbill et al. found in a survey of biliary endoscopists that 33% used straight stents, 30% used pigtail stents, and 35% used a combination. In addition, the survey found that internal flanges were always used in 14%, never used in 54%, and sometimes used in 32% [34]. Two randomized controlled prospective studies have compared the outcomes of a short straight 5 French stent without an inner flange with an unflanged long single pigtail 3 French stent. The study by Guda et al., published only in abstract form, found a higher placement failure rate in the 3 French group of 36 patients, a higher spontaneous dislodgement rate in the 5 French group of 43 patients, and a similar pancreatitis rate [35]. Meanwhile, Chahal et al. demonstrated a significantly higher placement failure rate (8.3% versus 0%; *P* = 0.0003), a nonsignificant higher pancreatitis rate (14% versus 9%; *P* = 0.3), and a lower spontaneous stent dislodgement rate (88% versus 98%; *P* = 0.0001) in the 3 French group of 133 patients compared with the 5 French group of 116 patients [36].

There is little data on the duration a pancreatic stent should remain in place to reduce the risk of pancreatitis. Sherman et al. found a significantly higher rate of pancreatitis in 46 patients in whom the pancreatic stent was removed immediately following needle-knife precut compared to 47 patients in whom the stent remained in-placed for 7–10 days (2.2% versus 21.3%; *P* = 0.004). Furthermore, pancreatitis developed in 13.8% of the 58 patients in whom the precut was performed without stent placement [24]. The optimal duration however is not known. One expert recommendation suggests that pancreatic stenting for a minimum of 24 hours in high-risk cases such as SOD should suffice. In contrast, pancreatic stenting for a few hours should be satisfactory in lower-risk cases such as those where biliary access is difficult [33].

*With regard to pancreatic stenting, pancreatic stent placement reduces the rate of post-ERCP pancreatitis in high-risk cases. Short 5 French stents are easier to deploy and more likely to migrate spontaneously compared with long 3 French stents. However, they do not confer a benefit in terms of pancreatitis risk reduction. The optimal duration for stents to remain in place is unknown.*

#### 6.2.5. Endoscopic Sphincterotomy

Thermal injury following application of electrosurgical current during biliary or pancreatic sphincterotomy has been implicated in the pathogenesis of post-ERCP pancreatitis [7, 37]. This is likely related to impaired drainage of the pancreatic duct from the resulting edema of the ampullary tissue. Pure current, in comparison to blended or “endocut” current, provides superior tissue cutting capability and, in theory, should be associated with less edema and a lower risk of pancreatitis. However, the incidence of bleeding is significantly higher when pure-cut current is used [38]. The type of current used for sphincterotomy and its association with pancreatitis have produced conflicting results. 

In a randomized controlled study involving 170 patients, Elta et al. demonstrated that the use of pure-cut current was associated with a lower incidence of pancreatitis compared with blended current (3% versus 12%; *P* < 0.05) [39]. This was further supported by randomized controlled trial by Stefanidis et al. (3.2% versus 12.9%; *P* = 0.048) [40]. In contrast, both MacIntosh et al. and Norton et al. reported in randomized controlled trials of 246 and 267 patients, respectively, no significant difference in the rate of pancreatitis between pure-cut and blended current (7.8% versus 6.1%; *P* = 0.62 [41], 0.7% versus 2.3%; *P* > 0.05 [42]). A subsequent meta-analysis of these 4 trials by Verma et al. found no significant difference in the pancreatitis rates between pure-cut and blended current (3.8% versus 7.9%) [38]. 

*There is no consensus on the type of current to be utilized during sphincterotomy to minimize the risk of post-ERCP pancreatitis.*

#### 6.2.6. Balloon Sphincteroplasty (Endoscopic Papillary Balloon Dilation)

Balloon sphincteroplasty or endoscopic papillary balloon dilation is a technique to use for biliary stone extraction used as an alternative to, or in conjunction with, endoscopic sphincterotomy. It has the advantage of preserving sphincter of Oddi function in younger patients [43], of lower bleeding rates compared with sphincterotomy [44], and of removing stones in Billroth II cases when sphincterotomy can be technically very challenging [45]. However, a multicentre randomized controlled trial found a significantly higher morbidity rate including pancreatitis following balloon sphincteroplasty in 117 patients compared to endoscopic sphincterotomy performed in 120 patients (15.4% versus 0.8%; *P* < 0.001) [46]. Indeed, there were 2 deaths due to pancreatitis following balloon sphincteroplasty and none following sphincterotomy. Furthermore, Baron and Harewood demonstrated in a meta-analysis of eight prospective randomized trials that post-ERCP pancreatitis occurred more commonly in the balloon dilation group (7.4% versus 4.3%, *P* = 0.05), leading the authors to conclude that it should be avoided in routine practice [44].

However, since the study by Baron and Harewood [44], a number of studies have demonstrated that balloon dilation following sphincterotomy can be used effectively and safely to extract bile duct stones. Maydeo and Bhandari demonstrated in a prospective study involving 60 patients that large diameter (12–15 mm) balloon dilation following endoscopic sphincterotomy did not result in any cases of postprocedure pancreatitis [47]. Furthermore, Heo et al. found no difference in the rate of pancreatitis in a prospective trial of 200 patients, equally randomized to either balloon dilation (12–20 mm) following sphincterotomy or sphincterotomy alone (4.0% in both groups) [48]. The safety of the combined procedure may be related to the force of the balloon exerted in the direction of the biliary sphincterotomy and away from the pancreatic orifice.

*Endoscopic papillary balloon dilation alone is associated with an unacceptably high risk of pancreatitis. This does not appear to be the case when it is performed in conjunction with endoscopic sphincterotomy.*

#### 6.2.7. Needle-Knife Precut

Precutting with a needle knife is typically used for access to the biliary system when standard cannulation techniques have been unsuccessful. This technique has been shown to be an independent risk factor for pancreatitis [4, 49]. However, the risk may be related more to the multiple cannulation attempts or pancreatic duct injections rather than the precut technique itself. This issue has been addressed in a number of randomized prospective trials. Manes et al. randomized 151 patients to either needle-knife precut (fistulotomy) or persistence with standard cannulation in cases of difficult biliary cannulation defined as unsuccessful cannulation after 10 minutes. The pancreatitis rate was significantly lower in the precut group (2.6 versus 14.9%; *P* = 0.008) [50]. A further study by Cennamo et al., where patients were randomized to either precutting (needle knife papillotomy) or persistence with standard cannulation after 5 minutes, found a similarly lower rate of pancreatitis in the precut group (3% versus 5%) [51]. A subsequent meta-analysis involving 6 studies demonstrated that early precut implementation significantly reduced the risk of post-ERCP pancreatitis when compared with standard cannulation (2.5% versus 5.3%, OR 0.47, 95% CI 0.24–0.91) [52].

There are a number of different approaches to performing needle-knife precut. The most widely performed precut techniques include needle-knife papillotomy, where the precut starts at the papillary orifice, and needle-knife fistulotomy, where the precut is superior to and separate from the papillary orifice. However, there is little high level evidence on the optimal needle-knife technique to use. Mavrogiannis et al. found in a randomized prospective study a lower rate of pancreatitis in 74 patients who underwent needle-knife fistulotomy compared to 79 patients who underwent needle-knife precut papillotomy (0% versus 7.59%, *P* < 0.05) [53]. Abu-Hamda et al. demonstrated a similar lower rate of pancreatitis in a retrospective series comparing the fistulotomy technique in 44 patients with the papillotomy technique in 47 patients (0% versus 12.8%; *P* = 0.03). While the authors comment on the retrospective nature and small sample size of the study, they highlight the post-ERCP pancreatitis can be best minimized by completely avoiding the papillary orifice [54].

*Early needle-knife precut implementation in cases of difficult biliary cannulation is associated with a lower risk of post-ERCP pancreatitis compared with persistence with standard cannulation techniques. Needle-knife fistulotomy technique may be superior to needle-knife papillotomy.*

#### 6.2.8. Sphincter of Oddi Manometry (SOM) and Sphincter of Oddi Dysfunction (SOD)

Sphincter of Oddi manometry (SOM) is the gold standard diagnostic test for sphincter of Oddi dysfunction (SOD). It is generally accepted to be associated with a relatively higher risk of pancreatitis. There are a number of methods that have been shown to reduce this risk. Early manometry was performed using continuous perfusion compared with more recent manometry which involves continuous aspiration of the perfused fluid, in theory reducing the risk of perfusion-related hydrostatic ductal injury. Sherman et al. found in a randomized controlled trial involving 76 patients a significant reduction in pancreatitis when manometry was performed with an aspirating catheter compared with a standard perfusion catheter (3.0% versus 23.5%; *P* = 0.01) [55]. Specific manometry of either the bile or pancreatic sphincter may also be an important contributory factor to pancreatitis development. Rolny et al. reported acute pancreatitis in 11% of patients who had pancreatic manometry alone compared with 1% who had biliary manometry alone [56]. Indeed, Sherman et al., in a further study, found no difference in pancreatitis rates in 36 patients randomized to biliary manometry with either an aspirating or a standard catheter, suggesting that perfusion injury may only be a problem when pancreatic manometry is performed [57]. Prophylactic pancreatic stent placement has also been shown to be of benefit in reducing pancreatitis in cases of SOD and following manometry. The initial randomized controlled trial of 80 patients with SOD documented by positive manometry demonstrated that stenting significantly reduced the rate of pancreatitis following biliary sphincterotomy compared with controls (7% versus 26%; *P* = 0.03) [24]. A subsequent study by Fazel et al. of 76 high-risk patients defined as having difficult cannulation, or undergoing manometry or endoscopic sphincterotomy, found a significantly lower frequency of pancreatitis in those who underwent pancreatic stenting compared to those who did not (5% versus 28%; *P* > 0.05) [29].

There is some evidence to support that the risk of pancreatitis may be more likely related to the underlying SOD and not the manometry per se. Firstly, the rates of pancreatitis in the manometry studies performed with an aspirating catheter by Sherman et al. [55, 57] are similar to those for ERCP in general. Furthermore, the multicentre study by Freeman et al. found a similar rate of pancreatitis between those who underwent biliary sphincterotomy with suspected SOD and those who underwent sphincterotomy in conjunction with manometry (20.3% versus 17.9%). Interestingly, severe pancreatitis was more common in patients who underwent sphincterotomy without manometry (3.6% versus 0.8%) [2]. In addition, a retrospective review of 100 consecutive patients demonstrated a significantly lower rate of pancreatitis in patients who had manometry only compared to those who had undergone both manometry and ERCP (9.3% versus 26.1%). Performance of sphincterotomy did not increase the risk beyond that associated with ERCP [58]. 

*Potential methods for reducing the rate of pancreatitis associated with sphincter manometry include performing pancreatic manometry with an aspirating catheter, performing biliary manometry alone in cases of suspected biliary disease, and placing prophylactic pancreatic stents. However, it should not be assumed that avoiding manometry in suspected SOD will reduce the risk of post-ERCP pancreatitis.*

#### 6.2.9. Endoscopic Ampullectomy

Endoscopic snare removal of the major duodenal papilla (endoscopic ampullectomy) has been advocated as a treatment for both adenomas that occur sporadically and in association with familial adenomatous polyposis [59].

**Postprocedure Pancreatitis**

A number of studies suggest that placement of a pancreatic stent reduces this risk. However, high-level evidence is lacking.

In a retrospective series of 16 patients by Zádorová et al., postampullectomy pancreatitis was reported in 0% and 20% of patients with and without a pancreatic stent, respectively [60]. Cheng et al. demonstrated in a further retrospective series of 55 patients that pancreatic stenting was associated with a lower, but not statistically significant, rate of pancreatitis (9.6% versus 25%; *P* = 0.33) [61]. In addition, a prospective trial by Harewood et al. found a significantly higher rate of pancreatitis in the 9 patients who did not undergo pancreatic stenting compared to the 10 patients who did (33% versus 0%; *P* = 0.02) [62]. However, this trial was stopped prematurely because of concerns of the risk of pancreatitis and did not reach the study's power calculation of 25 patients in each group.

*Although high-level evidence is not available, pancreatic stenting following endoscopic ampullectomy is recommended to reduce postprocedure pancreatitis.*

### 6.3. Pharmacological Agents

The ideal pharmacological agent should be highly effective in reducing post-ERCP pancreatitis, have a short administration time, be well tolerated with a low side-effect profile and cost-effective. Several agents have shown promise. However, the vast majority have fallen short of these goals (Table 3). 

#### 6.3.1. Nonsteroidal Anti-Inflammatory Drugs (NSAIDs)

NSAIDs are potent inhibitors of a number of inflammatory mediators including prostaglandins and phospholipase-A2, and both of which may play a role in the pathophysiology of acute pancreatitis [63]. Elmunzer et al. demonstrated in a meta-analysis, from four randomized controlled trials involving 912 patients, that prophylactic rectal NSAIDs were effective in reducing pancreatitis, with a pooled relative risk after administration of 0.36 (95% CI 0.22–0.60) [64]. In addition, no adverse events attributable to NSAIDs were reported. Two of the trials evaluated rectal diclofenac immediately after procedure, while the other two evaluated rectal indomethacin immediately preprocedure, and all four found a positive result in post-ERCP pancreatitis reduction. Interestingly, the randomized prospective trial by Cheon et al. found no difference in the 105 patients who received oral diclofenac compared with the 102 patients who received placebo (16.2% versus 16.7%; *P* = NS) [65]. Possible explanations for this difference may relate to peak plasma NSAID concentrations, which occur within 30 minutes with rectal administration in contrast to 2 hours with oral administration. Furthermore, bioavailability is reduced with oral administration because of first pass metabolism [66]. NSAIDs are relatively inexpensive and easy to administer as a once-off dose in comparison to other potentially promising agents which require continuous infusions and may not be readily available. Although routine rectal administration of 100 mg of diclofenac or indomethacin, immediately before or after ERCP, is recommended in the guidelines published by the European Society of Gastrointestinal Endoscopy, this practice has not yet been widely adopted [15]. 

#### 6.3.2. Glyceryl Trinitrate

Glyceryl trinitrate (GTN) is a smooth muscle relaxant which can lower basal pressure in the Sphincter of Oddi. It is most easily administered either by sublingual spray or transdermal patch. The results from single center prospective controlled trials of its effect on the reduction of post-ERCP pancreatitis are conflicting. Kaffes et al. found no benefit with the transdermal patch compared with placebo in 318 patients (7.7% versus 7.4%; *P* = NS) [67], while Moretó et al. found a significant reduction in pancreatitis in 144 patients (15% versus 4.2%; *P* < 0.05) [68]. The conclusions drawn from a number of meta-analyses are similar. Bai et al. found, from 8 randomized controlled trials involving 1920 patients, that the incidence of pancreatitis was significantly reduced by GTN treatment compared with placebo (5.9% versus 9.8%; *P* = 0.002) [69]. In contrast, both meta-analyses by Bang et al. and Shao et al. did not show an overall significant reduction in post-ERCP pancreatitis [70, 71]. In addition to a benefit of GTN shown in some studies in the prophylaxis of post-ERCP pancreatitis, it is inexpensive, easy to administer, and has few major side effects. However, the optimal dose, timing, and route of administration require further clarification. Currently, it is not recommended for routine use in ERCP [15].

#### 6.3.3. Ceftazidime

There is only one study which has evaluated a possible role for antibiotics in the prevention of post-ERCP pancreatitis. This prospective randomized controlled trial demonstrated that 2 g of the cephalosporin, ceftazidime administered intravenously 30 minutes before ERCP, significantly reduced the incidence of post-ERCP pancreatitis in the control group of 160 patients compared with the antibiotic group of 155 patients (9.4% versus 2.6%; *P* = 0.009) [72]. However, the quality of the study is questionable as the control group received no antibiotics rather than placebo. There have been no confirmatory studies on the use of antibiotics.

#### 6.3.4. Somatostatin and Octreotide

Both somatostatin and its synthetic analogue, octreotide, are potent inhibitors of exocrine secretion of the pancreas, which play an important role in the pathogenesis of acute pancreatitis by causing autodigestion of the organ [73]. 

Two meta-analyses analyzed the efficacy of somatostatin for the prophylactic management of post-ERCP pancreatitis. Andriulli et al. included results from 9 studies and found a nonsignificant effect of somatostatin on pancreatitis (7.3% of controls versus 5.3% of treated patients; OR 0.73; 95% CI 0.54–1.006). Furthermore, this meta-analysis produced nonbeneficial results for both short- (<6 hours) and long-term (≥12 hours) somatostatin infusions (6.4% in controls versus 8.5% in treated patients; OR 1.361, 95% CI 0.886–2.091, 6.4% in controls versus 3.0% in treated patients; OR 0.447, 95% CI 0.133–1.508, resp.) [74]. Rudin et al. also demonstrated in a meta-analysis involving 3,130 patients from 7 studies that a short-term infusion (<12 hours) was not beneficial. However, this meta-analysis yielded a significant risk reduction of 7.7% for long-term somatostatin infusion (≥12 hours) [75].

Both meta-analyses included the same studies that looked at bolus administration of somatostatin prior to ERCP and found a significant reduction in post-ERCP pancreatitis rates (11.3% in controls versus 3.0% in treated patients; OR 0.271, 95% CI 0.138–0.536). However, the pancreatitis rate of the control patients in the bolus group was twice that of the control patients in both the short- and long-term infusion groups (11.3% versus 6.4% and 6.4%, resp.). This led the authors to conclude that caution should be applied when bolus administration of somatostatin is being considered [74].

Octreotide is a synthetic analogue with a longer half-life than somatostatin.The results from studies have produced conflicting results. Thomopoulos et al. demonstrated a significant reduction in the incidence of pancreatitis between octreotide (1.5 mg subcutaneously in three divided doses) 24 hours prior to ERCP and placebo in a multicentre randomized controlled trial involving 202 patients (2.0% versus 8.9%; *P* = 0.03) [76]. In contrast, Testoni et al. demonstrated no difference in 114 patients randomized to either octreotide (0.6 mg subcutaneously in three divided doses) 24 hours prior to ERCP or placebo (12.0% versus 14.3%; *P* = NS) [77]. A subsequent meta-analysis of 15 studies found that octreotide was not beneficial in the prevention of post-ERCP pancreatitis [78]. However, a more recent meta-analysis involving 18 studies demonstrated that octreotide used at a dose of at least 0.5 mg significantly reduced the rate of post-ERCP pancreatitis compared with controls (3.4% versus 7.5%; *P* = 0.001). No benefit was identified when it was used at a lower dose (7.2% versus 6.0%; *P* = 0.35) [79]. The authors also concluded that there were insufficient data on the optimal timing and route of administration. Furthermore, the ESGE guidelines do not recommend octreotide for the prophylaxis of post-ERCP pancreatitis but comment that future studies should evaluate its efficacy at 0.5 mg or higher [15].

#### 6.3.5. Protease Inhibitors

One of the initial events in the development of acute pancreatitis is intracellular activation of trypsin. Protease inhibitors prevent activation of trypsin and have been used for both the treatment of acute pancreatitis and for the prevention of post-ERCP pancreatitis. These include gabexate, ulinastatin, and nafamostat mesylate. The published evidence on a potential benefit of these agents in post-ERCP pancreatitis comes from high-level randomized controlled trials but has produced conflicting results.

Two such prospective randomized controlled trials have shown a benefit for the use of gabexate in the reduction of post-ERCP pancreatitis. Xiong et al. demonstrated a significant reduction in 97 patients treated with gabexate, commencing 30 minutes prior to ERCP and continuing for 4 hours after, compared to 96 patients treated with placebo (3.1% versus 10.5%; *P* = 0.40) [80]. Manes et al. found a similar reduction regardless of whether gabexate was administered pre- or post-ERCP (3.9% in group given gabexate 1 hour pre, versus 3.4% in group given gabexate 1 hour post, versus 9.4% in placebo group; *P* < 0.01) [81]. In contrast, Andriulli et al. demonstrated in two separate large multicentre trials that both short (2 hours) and long term administration (>6.5 hours) of gabexate was ineffective at reducing post-ERCP pancreatitis compared with placebo (6.5% versus 8.1%; *P* = NS, 4.8% versus 5.8%; *P* = NS) [82, 83]. A subsequent meta-analysis incorporating 5 studies reported that gabexate was ineffective for the prevention of post-ERCP pancreatitis [74].

One of the major drawbacks associated with gabexate is its short half-life of 55 seconds and hence the need for an infusion over several hours. In contrast, ulinastatin has a longer half-life of 35 minutes and can be given as a bolus injection [84]. Tsujino et al. found in a randomized, prospective trial, involving 406 patients, that ulinastatin (150,000 U) administered prior to ERCP significantly reduced the incidence of post-ERCP pancreatitis compared with placebo (2.9% versus 7.4%, *P* = 0.041) [85]. However, routine prophylactic use of ulinastatin prior to ERCP is unlikely to be cost-effective because the frequency of post-ERCP pancreatitis is low and the majority of cases are mild. With this in mind, Yoo et al. randomized 227 patients, identified during the ERCP to be at high risk of post-ERCP pancreatitis development, to either ulinastatin (100,000 U) or placebo immediately after the procedure and found no significant reduction in the treatment group (5.6% versus 6.7%; *P* = 0.715) [86]. This study was included in a recent meta-analysis of 7 randomized trials which demonstrated that ulinastatin reduced the incidence of post-ERCP pancreatitis (OR 0.53; 95% CI 0.31–0.89; *P* = 0.02) and subsequently concluded that ulinastatin was of value when administered prior to ERCP at a dose not less than 150,000 U to patients at average risk of developing pancreatitis [87].

To date, two prospective randomized controlled single-center trials have shown the benefit of nafamostat in the prevention off post-ERCP pancreatitis [88, 89]. Choi et al. demonstrated a post-ERCP pancreatitis rate of 3.3% in the 354 patients treated with nafamostat compared with 7.4% in the 350 patients treated with placebo, commencing 1 hour before and continuing for 24 hours after ERCP (*P* = 0.018). Similarly, Yoo et al. found a significant reduction in the 286 patients equally randomized to either nafamostat or placebo, commenced 60 minutes prior to and continuing for hours after ERCP (2.8% versus 9.1%; *P* = 0.03). Despite these positive results, the length of infusion and routine prophylactic use are impractical. Further studies are required to determine if bolus injection and post-procedural administration in high-risk patients produce a similar risk reduction.

Protease inhibitors have shown some promise. However, they are costly and may require hospital admission because of duration of administration postprocedure, and, as a recent meta-analysis shows, the numbers needed to treat to prevent a single episode of post-ERCP pancreatitis are extremely high (gabexate = 33.3 and ulinastatin = 28.6) [90]. 

## 7. Allopurinol

Capillary endothelial injury, mediated by oxygen-derived free radicals, may be involved in the pathogenesis of acute pancreatitis [91, 92]. Xanthine oxidase catalyzes the conversion of hypoxanthine to xanthine, which generates oxygen-derived free radicals.

Allopurinol is a xanthine oxidase inhibitor. Marks et al. initially demonstrated in an animal model that pretreatment with oral allopurinol decreased the incidence of ERCP-induced pancreatitis [93]. The results from subsequent human studies have been conflicting. Both Katsinelos et al. and Martinez-Torres et al. demonstrated, in prospective placebo-controlled trials of 243 and 170 patients, respectively, a benefit for its use in the prevention of post-ERCP pancreatitis [94, 95]. In the former, patients received 600 mg dose at 15 and 3 hours prior to ERCP with a subsequent significant reduction in post-ERCP pancreatitis compared with placebo (3.2% versus 17.8%; *P* < 0.001), while in the latter, patients received 300 mg at the same timing with a similar significant reduction compared with placebo (2.3% versus 9.4%; *P* = 0.04). In contrast, Mosler et al. found in a prospective randomized trial of 701 patients no difference between allopurinol and placebo administered at 4 hours and 1 hour preprocedure (12.96% versus 12.14%; *P* = 0.52) [96]. In addition, Romagnuolo et al. did not demonstrate a significant reduction in post-ERCP pancreatitis rates in 586 patients randomized to either 300 mg allopurinol or placebo 1 hour prior to ERCP (5.5% versus 4.1%; *P* = 0.44) [97]. The conflicting results from these studies may suggest that both the dose and timing of administration of allopurinol may influence the development of post-ERCP pancreatitis. However, a subsequent meta-analysis incorporating 6 randomized controlled trials and 1554 patients demonstrated that prophylactic allopurinol did not reduce the frequency or severity of post-ERCP pancreatitis and led the authors to conclude that allopurinol should not be recommended for the prophylactic prevention of post-ERCP pancreatitis [98].

### 7.1. Corticosteroids

In a prospective randomized controlled multicentre study of 1115 patients, prophylaxis with 40 mg of oral prednisone did not alter either the frequency (16.6% in the prednisone group versus 13.6% in the placebo group; *P* = 0.19) or the severity of pancreatitis compared with placebo [99].

### 7.2. Heparin

Heparin has an inhibitory effect on proteases in both plasma and pancreatic tissue and also improves pancreatic microcirculation during experimental pancreatitis [100]. It has been suggested as a potential treatment in the prevention of post-ERCP pancreatitis. However, a prospective randomized controlled multicentre study demonstrated that subcutaneous low molecular weight heparin in 221 patients offered no benefit compared to placebo in 227 patients in terms of reduction of pancreatitis (8.1% versus 8.8%; *P* = 0.87) [101].

### 7.3. N-Acetylcysteine

N-acetylcysteine is a free radical scavenger and has been shown to decrease the incidence and severity of experimental pancreatitis [102]. However, two randomized controlled trials have not shown its benefit in the prevention of post-ERCP pancreatitis. Both Katsinelos et al. [103] and Milewski et al. [104] found no difference in pancreatitis rates in 249 patients (12.1% versus 9.6%; *P* > 0.05) and 106 patients (7.3% versus 11.8%; *P* = NS) randomized to N-acetylcysteine or placebo, respectively.

*While a number of agents have shown promise in clinical trials, there is currently no accepted pharmacologic intervention to prevent post-ERCP pancreatitis. However, this continues to be an active area of research.*

## 8. Conclusions

Awareness of both patient- and procedure-related factors for the development of post-ERCP pancreatitis can be used to risk stratify patients in particular to identify those in which pharmacological or procedural interventions should be considered. 

ERCP should be avoided in unnecessary or low yield cases especially when multiple patient-related risk factors for the development of pancreatitis are present. A number of pharmacological agents, in particular rectal NSAIDs, have also shown promise, but none are currently being consistently used. The procedural interventions that have been demonstrated to reduce the incidence of post-ERCP pancreatitis including guide-wire cannulation rather than contrast injection, and pancreatic stent placement in high-risk cases.

## Figures and Tables

**Table 1:** Risk factors associated with the development of post-ERCP pancreatitis. Risk factors, apart from ampullectomy, are significant by multivariate analyses in prospective multicenter studies and by meta-analysis [3–6]. Ampullectomy is generally accepted to be a risk factor for pancreatitis. SOD: sphincter of Oddi dysfunction.

Patient-related factors	Procedure-related factors	Operator-related factors
Female	Precut sphincterotomy	Trainee involvement
SOD	Pancreatic duct injection	
Previous pancreatitis	Balloon dilation of intact sphincter	
Chronic pancreatitis absent	Pancreatic sphincterotomy	
Younger age (<60 years)	Difficult cannulation	
Normal bilirubin	Minor papilla sphincterotomy	
	Pain during ERCP	
	Ampullectomy	

**Table 2:** Studies demonstrating effect of pancreatic stenting on post-ERCP pancreatitis. Difficult cannulation was defined as that requiring greater than 30 minutes of manipulation to achieve successful cannulation.

Study	Study no.	Rate of pancreatitis		*P* value	Indications for pancreatic stent placement				
		No-stent group	Stent group		SOD	Precut	Difficult cannulation	Balloon dilation	Pancreatic sphincterotomy
Smithline et al. [23]	93	18%	14%	0.60	+	+			
Sherman et al. [24]	104	21%	2%	0.004		+			
Elton et al. [25]	164	12.5%	0.7%	0.003					+
Tarnasky et al. [26]	80	26%	7%	0.03	+				
Patel et al. [27]	36	33%	11%	<0.05	+				
Aizawa and Ueno [28]	130	6%	0%	0.11				+	
Fazel et al. [29]	74	28%	5%	0.009	+		+		
Sofuni et al. [30]	211	13.6%	3.2%	0.019	All consecutive ERCPs irrespective of specific risk factors				
Tsuchiya et al. [31]	64	12.5%	3.1%	>0.05	All consecutive ERCPs irrespective of specific risk factors				

**Table 3:** Pharmacological agents that have been used in the prevention of post-ERCP pancreatitis.

*Agents with proven efficacy*	
Non steroidal anti-inflammatory drugs	
Diclofenac	
*Agents with possible efficacy*	
Ceftazidime	
Glyceryl trinitrate	
Octreotide	
Protease inhibitors	
Ulinastatin	
Nafamostat	
Somatostatin	
*Agents with proven inefficacy*	
Allopurinol	
Corticosteroids	
Heparin	
N-acetylcysteine	
Protease inhibitor	
Gabexate	
